# The impact of dextran sodium sulphate and probiotic pre-treatment in a murine model of Parkinson’s disease

**DOI:** 10.1186/s12974-020-02062-2

**Published:** 2021-01-09

**Authors:** Zach Dwyer, Melany Chaiquin, Jeffrey Landrigan, Kiara Ayoub, Pragya Shail, Julianna Rocha, Christie L. Childers, Kenneth B. Storey, Dana J. Philpott, Hongyu Sun, Shawn Hayley

**Affiliations:** 1 Department of Neuroscience, Carleton University, 1125 Colonel By Drive, Ottawa, Ontario K1S 5B6 Canada; 2 Department of Immunology, University of Toronto, Toronto, Ontario M5S 1A8 Canada; 3 Institute of Biochemistry and Department of Biology, Carleton University, 1125 Colonel By Drive, Ottawa, Ontario K1S 5B6 Canada

**Keywords:** Microglia, Probiotic, Inflammatory neurodege5neration, Microbiota

## Abstract

**Background:**

Recent work has established that Parkinson’s disease (PD) patients have an altered gut microbiome, along with signs of intestinal inflammation. This could help explain the high degree of gastric disturbances in PD patients, as well as potentially be linked to the migration of peripheral inflammatory factors into the brain. To our knowledge, this is the first study to examine microbiome alteration prior to the induction of a PD murine model.

**Methods:**

We presently assessed whether pre-treatment with the probiotic, VSL #3, or the inflammatory inducer, dextran sodium sulphate (DSS), would influence the PD-like pathology provoked by a dual hit toxin model using lipopolysaccharide (LPS) and paraquat exposure.

**Results:**

While VSL #3 has been reported to have anti-inflammatory effects, DSS is often used as a model of colitis because of the gut inflammation and the breach of the intestinal barrier that it induces. We found that VSL#3 did not have any significant effects (beyond a blunting of LPS paraquat-induced weight loss). However, the DSS treatment caused marked changes in the gut microbiome and was also associated with augmented behavioral and inflammatory outcomes. In fact, DSS markedly increased taxa belonging to the Bacteroidaceae and Porphyromonadaceae families but reduced those from Rikencellaceae and S24-7, as well as provoking colonic pro-inflammatory cytokine expression, consistent with an inflamed gut. The DSS also increased the impact of LPS plus paraquat upon microglial morphology, along with circulating lipocalin-2 (neutrophil marker) and IL-6. Yet, neither DSS nor VSL#3 influenced the loss of substantia nigra dopamine neurons or the astrocytic and cytoskeleton remodeling protein changes that were provoked by the LPS followed by paraquat treatment.

**Conclusions:**

These data suggest that disruption of the intestinal integrity and the associated microbiome can interact with systemic inflammatory events to promote widespread brain-gut changes that could be relevant for PD and at the very least, suggestive of novel neuro-immune communication.

## Introduction

Parkinson’s disease (PD) is characterized by a loss of substantia nigra pars compacta (SNc) dopaminergic neurons resulting in motor disturbances [1, 2]. Both genetic and environmental factors likely interact to provoke the disease and neuroinflammatory factors have been implicated in such interactions [3, 4]. It could be that environmental insults (such as pesticides, heavy metals, pathogens, or even psychological stress) can act as triggers that induce a pro-inflammatory state [5]. Indeed, combinations of genetic factors, including LRRK2 or the alpha-synuclein A53T mutation, together with environmental toxins are common models of PD [6–8]. Regardless of the model utilized, microglial cells, the resident immune cells of the central nervous system (CNS) have been repeatedly implicated in PD pathology [9–11]. Likewise, peripheral cells of the innate and adaptive immune system may be involved in the spread of PD pathology. In fact, some suggest that initial PD pathology might actually originate outside the brain, possibly in the gut and associated cells [12–14]. Recent studies do report that PD patients have altered gut microbiota [15–17] and the composition of the gut microbiota may be of special significance given its role in modulating peripheral immune cells.

While gastrointestinal difficulties have long been known to be common in PD, recent evidence suggests that an irritable bowel and constipation in middle age actually increases the risk of developing PD [18, 19]. Links between Crohn’s disease and PD have also been recently uncovered with the gene, LRRK2, being suggested to be a common mechanistic inflammatory factor shared by these two seemingly disparate diseases [20, 21]. Other studies have found increased alpha-synuclein load in the gut of PD patients [22, 23] and one clinical study found that truncal vagotomy reduced PD risk later in life [12]. In 2016, Sampson et al*.* published a study demonstrating that germ free alpha-synuclein overexpressing (ASO) mice showed reduced pathology at 14 months, whereas ASO mice harboring bacteria isolated from PD patients showed earlier symptom onset and enhanced pathology [24]. A further study found that orally administered rotenone, a pesticide implicated in PD, altered the gut microbiota and led to early accumulation of alpha-synuclein in the gut tissues [25].

Little success has been found regarding the beneficial effects of probiotics, and indeed, striking differences are evident even with probiotics with the same formulation [26, 27]. VSL#3 is a probiotic consisting of eight cultured bacteria (Lactobacillus plantarum, Lactobacillus delbrueckii subsp. Bulgaricus, Lactobacillus paracasei, Lactobacillus acidophilus, Bifidobacterium breve, Bifidobacterium longum, Bifidobacterium infantis, and Streptococcus salivarius subsp. Thermophilus) which is currently prescribed for irritable bowel syndrome and has been shown to modulate intestinal permeability [28, 29]. Recent papers have also found that VSL#3 may play a protective role in visceral hypersensitivity [30] and renal ischemia injuries [31] by modulating immune cell phenotypes. In contrast, agents that irritate the gut and cause inflammation are thought to negatively affect the microbiome and could possibly contribute to PD. Dextran sodium sulphate (DSS), which is used as a common model of colitis, has marked effects upon intestinal integrity and microbial constituents. DSS is known to particularly damage epithelial cells of the intestine and promote a leaky mucosal barrier [32]. DSS-treated mice displayed infiltration and activation of inflammatory neutrophils, macrophages, and T lymphocytes, together with elevations in circulating inflammatory cytokines [32, 33]. There is also an overall shift in the quantity and diversity of the microbiota species, with DSS destabilizing the microbiota [34].

To our knowledge, no studies have attempted to alter the existing microbiome prior to the introduction of a toxin-based model of PD. Hence, we assessed the impact of pre-exposure to either VSL#3 or DSS upon outcomes induced by a multi-hit LPS plus paraquat model of PD. We used this model since these toxicants may interact with peripheral processes (i.e., altered gut) and the use of two “hits” from different challenges may be more relevant to the complex origins of the disease. Paraquat also has some degree of ecological relevance since it is still used in agriculture and has been epidemiologically linked to PD in the community and we previously found that paraquat can induce stressor effects (e.g., activation of the HPA axis) and promote behaviors that are often co-morbid with Parkinson’s (e.g., depressive-like symptoms) [35–38]. Overall, our data support the notion that an inflamed intestine and accompanying changes in microbiota can have widespread effects upon inflammatory factors within the brain and blood, and behavioral symptoms, but did not influence the loss of dopaminergic neurons, nor were there any protective effects of the probiotic.

## Methods

### Animals

**Fig. 1:**
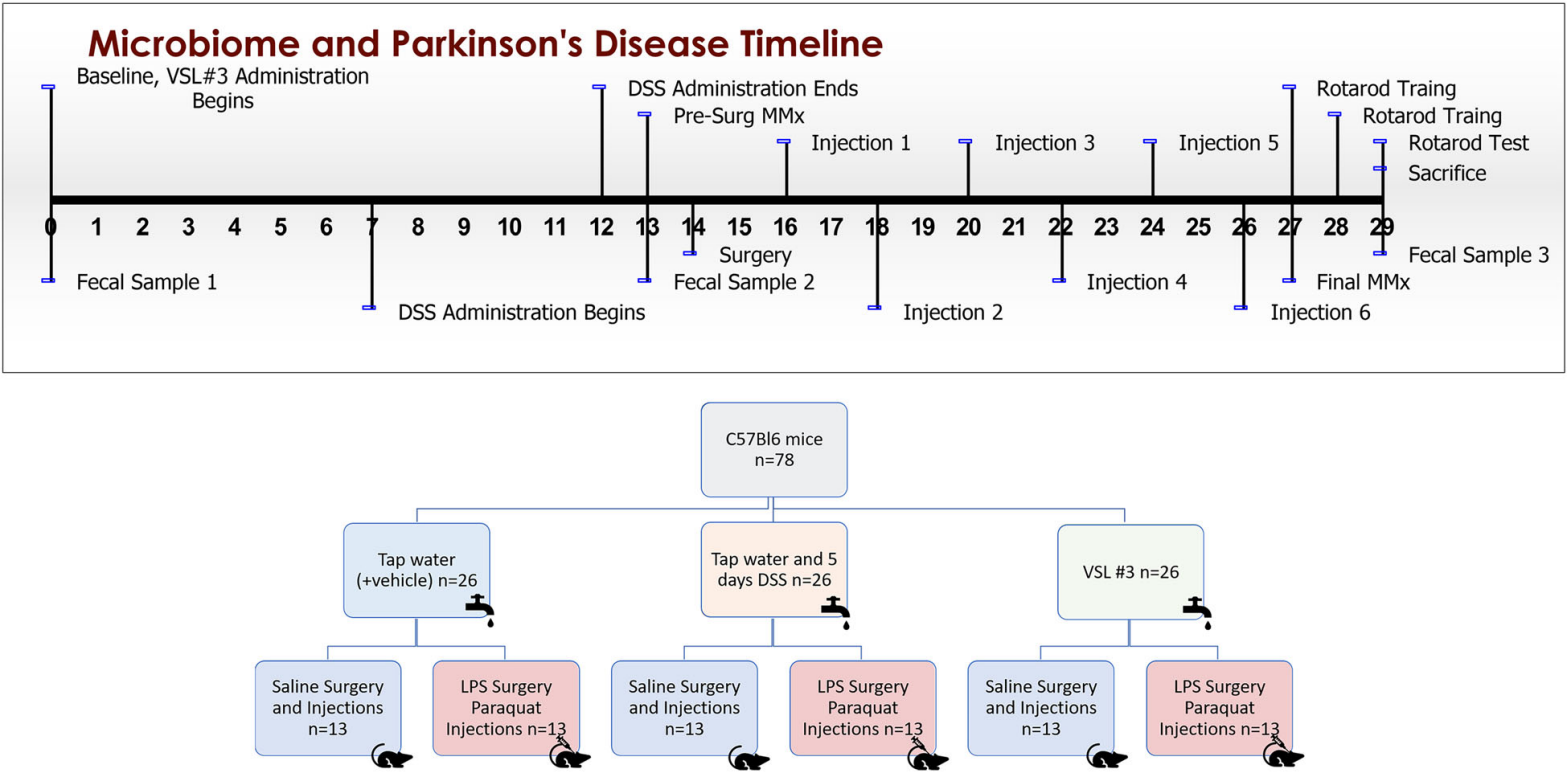
Timeline of experimental treatments, behaviors, and sample collections.

Seventy-eight C57Bl6/J male mice were purchased from Charles River laboratory at 8–10 weeks of age and single housed in standard cages upon arrival at Carleton University. Animals were single-housed in order to allow for accurate monitoring of daily solution consumption and to reduce unnecessary handling during weighing/injections, as well as preventing fighting, which can cause wounding confounds in toxin-treated mice. All animals were fed Harlan Labs 2014 rodent chow ad libitum for the duration of the experiment and housed under a normal 12-h light cycle. Upon arrival, animals were randomly assigned into one of three groups based on drinking water composition: (1) VSL #3, (2) DSS, or (3) tap water. Animals in the VSL #3 group were given 5.4 x 10^9^ CFU/day of VSL #3 dissolved in non-sterile tap water from day of arrival until sacrifice (Fig. 1). Animals in the DSS group were given water for the first 7 days followed by 250 mg/mL DSS for 5 days and then non-sterile tap water with cornstarch vehicle for the remainder of the experiment. Animals in the tap water control group were given non-sterile tap water with cornstarch vehicle freely throughout the experiment.

### VSL #3

Each morning one packet of freeze dried, unflavored VSL#3 (450 x 10^9^ CFU), commercially purchased from Ferring Canada, was dissolved in 250 mL of room temperature non-sterile tap water. Ten milliliters was then placed into each individual water tube. Tubes were labeled, weighed, and given to animals. When tubes were replaced the following day with fresh VSL#3, they were again weighed and the difference taken to determine the quantity ingested. Animals drank an average of 3 mL a day, and this did not differ between the groups. Thus, all mice received (1.8 x 10^9^ CFU/ml x 3 ml) approximately 5.4 x 10^9^ CFU per day. This dosage is well within the range of previous studies that used a mouse model of colitis; 4 x 10^9^ CFU/dose over a 7-day interval or 3 × 10^8^ CFU VSL#3 for a period of 28 days [39, 40]. But these studies used oral gavage, whereas we presently avoided using oral gavage owing to its inherent stressful impact.

### Surgeries

Two weeks after arrival, at 3 months of age, all animals underwent stereotaxic surgery. Animals were anesthetized with 5% isoflurane, weighed, and then subcutaneously administered 0.3 mL of saline and 20 mg/kg of the analgesic Tramadol. A 22-gauge injector was used to infuse half of each group with 2 μL of either saline or 1 μg/μL LPS directly above the substantia nigra pars compacta (SNc) and 4 mm below the surface of the skull. A Harvard Apparatus syringe pump was used to ensure a constant infusion over 4 min. The injector was left in place for 5 min after the infusion to allow the LPS/saline to absorb into the tissue before slowly being removed. Animals were given hydrogel for 4 days after surgery and 20 mg/Kg Tramadol subcutaneously twice a day for 3 days following surgery.

### Injections

Beginning 48 h after surgery, each animal received either 10 mg/kg of paraquat or an equivalent volume of saline through an intraperitoneal injection. Animals who received LPS during surgery received paraquat, while saline animals again received saline. Paraquat was freshly made each morning, animals were weighed, and these injections were given immediately after every 48 h for 11 days totaling six injections.

### Sacrifice

Immediately following the final behavioral testing session (rotarod) on Day 29, animals were sacrificed. Half received 0.15 mL of intraperitoneal sodium pentobarbital and blood was flushed using 5 mL of saline, followed by fixation with 40 mL of 4% paraformaldehyde. Twenty-four hours later, the brains were transferred to 10% sucrose and then transferred to 30% sucrose 48 h after sacrifice. The remaining half of the mice were rapidly decapitated, the animal’s trunk blood was collected into tubes containing 10 ul of 10% EDTA. The blood was then spun in a pre-chilled centrifuge at 2000 g for 20 min, and the resulting plasma was collected and flash frozen to − 80 °C. The brains of these mice were extracted and sectioned, and the hippocampus, anterior striatum, and SNc were punched for western blotting within 3 min of decapitation. Concurrently, the abdominal cavities were opened and the large intestine was removed and formed into a Swiss roll. This was cut in half; one side was paraffin-embedded and one side was flash frozen for qPCR.

### Behavioral analyses

#### Home-cage locomotor activity

Spontaneous home cage locomotor activity was measured over a complete 12-h light/dark cycle using a Micromax (MMx) infrared beam-break apparatus (Accuscan Instruments, Columbus, OH, USA), as previously described [41]. Activity assessment was completed following a 30-min acclimation period in a behavioral testing room following nestlet removal. Measurements of home-cage locomotor activity occurred once at baseline (Day 0), then again, the evening of the 2nd and 5th paraquat/saline injections.

#### Rotarod

On Day 27, animals began training on a rotarod apparatus (EzRod, Accuscan Instruments, Columbus, OH), which consists of a rubber coated horizontal beam 30 cm above the ground. Time to stay on the rotating beam provides an index of motor coordination. Animals received 2 days of training; on the first day, they were placed onto the beam for 5 min at 12 rpm and replaced every time they fell within the 5 min. This was repeated 1 h later and again after a further hour for a total of three training sessions. On Day 28, animals received their second rotarod training. The speed was increased to 22 rpm, but all other parameters remained the same. Test day occurred on Day 29, when the animals were placed on the rotarod for 3 sessions an hour apart in which the speed of the rod increased from 2 rpm to 44 rpm over the course of 5 min. The speed and time at which each animal fell was recorded.

#### Microbiome sequencing

A subset of fecal samples was extracted using a Fecal DNA Extraction Kit (Norgen Biotek). These samples were quantified using a nanodrop to assess DNA yield and quality and sent to Dalhousie’s Integrated Microbiome Resource (Dalhousie University, Halifax, NS) for 16S V4-V5 ribosomal sequencing.

#### Plasma lipocalin assay

Trunk blood was collected at time of decapitation and prepared as for the corticosterone assay in a separate aliquot at − 80 °C. Lipocalin-2 (LCN2) levels were determined by commercially available ELISA kit (R&D Systems, NE, USA, Cat #DY1857) following manufacturer’s instructions. Plasma samples were diluted 1:10,000 in assay buffer and assayed in duplicate within a single run. The intra-assay variability was less than 10%.

#### Plasma determination of cytokines

Trunk blood was collected at time of decapitation and prepared as for the corticosterone assay in a separate aliquot at − 80 °C. IL-6, TNF-α, IL-1B, and IL-10 levels were determined using a Luminex Immunoassay (R&D Systems, NE, USA) ran following kit instructions on a Luminex Magpix (Luminex Corporation, TX, USA). Samples were assayed in duplicate within a single run to control for inter-assay variability; the intra-assay variability was less than 10%.

#### Assessment of intestinal cytokine mRNA

##### RNA isolation

Approximately 50 mg of gut tissue was briefly homogenized using a Polytron PT1200 homogenizer in 0.5 mL of Trizol (Invitrogen, Carlsbad, CA, USA). Samples were sonicated for 30 s before 200 ul of chloroform were added, and samples were centrifuged for 15 min at 1200 rpm at 4 °C. The supernatant was transferred to microcentrifuge tubes, and samples were mixed with 500 ul of isopropanol and left on ice for 10 min to allow RNA precipitation. Samples were centrifuged for 15 min at 1200 rpm at 4 °C. The upper aqueous phase was discarded, and the pellets were washed with 1 ml of 70% ethanol, after which the samples were further centrifuged for 5 min at 7500 rpm at 4 °C. The supernatant was aspirated and pellets were air-dried for 15 min and then resuspended in 25 ul of RNase-free water. RNA concentration and quality were assessed by measuring the 260/280 nm ratio (> 1.8) using a Take3 micro-volume quantification plate (BioTek) and a powerwave HT spectrophotometer (BioTek). Total RNA integrity was determined by running RNA isolates on a 1% agarose gel stained with SYBR Green and verifying the bands for 28S and 18S ribosomal RNA.

##### cDNA synthesis

Oligo-dT primer ligation and reverse transcription was performed as described previously [42]. In brief, first-strand synthesis was performed using 1 ug of total RNA diluted in autoclaved RNase-free water to obtain a final volume of 10 ul. One microliter of 200 ng/ul oligo (dT) (5′-TTTTTTTTTTTTTTTTTTTTTV-3′; V = A or G or C; Sigma Genosys) was added to the samples, and samples were incubated in a thermocycler (Mastercycler Eppendorf) at 65 °C for 5 min, after which they were chilled on ice for 5 min. Samples were then incubated at 42 °C for 45–60 min in an Eppendorf thermocycler (Mississauga, ON, Canada) with 4 μl of 5× first-strand buffer (Invitrogen, Carlsbad, CA, USA), 2 μL of 0.1 M DTT (Invitrogen, Carlsbad, CA, USA), 1 μl of 10 mM dNTPs (BioShop, Burlington, ON, Canada), and 1 μl MMLV Reverse Transcriptase (Invitrogen, Carlsbad, CA, USA). Serial dilutions of the cDNA were prepared and stored at 4 °C until use.

##### qRT-PCR amplification

qRT-PCR assays were performed using a Bio-RAD MyiQ2 Detection System (BioRad, Hercules, CA, USA) following MIQE guidelines [43]. Twenty microliters of reactions were used, each consisting of 2 μl cDNA, 2 μl qRT-PCR buffer (100 mM Tris–HCl [pH 8.5], 500 mM KCl, 1.5% Triton X-100, 20 mM MgCl2, 2 mM dNTPs, and 100 nM fluorescein), 0.16 μl of 25 mM dNTPs, 4 μl of 1 M trehalose, 0.5 μl of 100% formamide, 0.1 μl of 100× SYBR Green diluted in DMSO, 0.5 μl of 0.3 nmol/μl forward primer, 0.5 μl of 0.3 nmol/μl reverse primer, 0.125 μl of 5 U/μl Taq Polymerase (BioShop, Burlington, ON, Canada), and 10.115 μl DEPC-treated water. The optimized PCR protocol consisted of an initial denaturing step at 95 °C for 2 min, followed by 60 cycles of 95 °C for 45 s, 57 °C annealing temperature for 45 s, and 72 °C for 45 s, and a final step of 72 °C for 4 min. All PCR runs underwent melt-curve analysis and dilution curve testing.

##### Data analysis

Raw cycle threshold (Ct) values obtained from each PCR run were converted to a linear form using 2^-C t^ calculations and were normalized against the reference gene, GAPDH. GAPDH was used as a reference gene as it exhibited stable expression levels in all conditions. The Ct of each mRNA was therefore normalized to the Ct of GAPDH from the same sample. The comparative ΔΔCt method was used to quantitate relative expression of mRNA expression [44].

### Western blot

Brain tissue punches and organs were collected to detect levels of GFAP and WAVE2, as previously described previously [45]. Briefly, whole cell lysates were homogenized in Radio Immuno Precipitation Assay (RIPA) buffer [50 mM Tris (pH 8.0), 150 mM sodium chloride, 0.1% sodium dodecyl sulphate (SDS), 0.5% sodium deoxycholate, and 1% Triton X-100] mixed with 1 tablet of Complete Mini ethylenediaminetetraacetic acid (EDTA)-free protease inhibitor (Roche Diagnostics, Laval, QC, Cat #11 836 170 001) per 10 mL of buffer. On the first day of analysis, proteins were separated using sodium dodecyl sulphate-polyacrylamide gel electrophoresis (SDS-PAGE). In order to determine total protein, membranes were incubated in REVERT total protein solution for a period of 5 min followed by placement into a REVERT wash solution (6.7% glacial acetic acid, 30% methanol, in water) two times for 2 min each. The membranes were then quickly rinsed with distilled water and imaged on our LI-COR Odyssey imaging system on the 700 channel for an exposure period of 2 min. Membrane incubation with mouse anti-GFAP (1:2000) and WAVE2 (1:4000) for a period of 60 min in 0.05% fish gelatin in TBS with 0.1% tween followed by 1 h in infrared anti-mouse conjugate at a concentration of 1:20,000 in 0.5% fish gelatin solution containing 0.2% tween and 0.01% SDS. Any unbound antibody was removed using 15 mL of TBS-T/membrane, and membranes washed and read on our Licor Odyssy system at the appropriate wavelength for 8 min.

### Immunohistochemistry

In order to examine microglial reactivity, sections were stained with ionized calcium-binding adapter molecule 1 (IBA1), a microglial marker found across the membrane. To assess dopamine neuronal survival, tyrosine hydroxylase (TH) immunostaining was used. The brains were sliced into 40-um thick sections on a Shandon AS620 cryostat (Fisher Scientific), and sections were immediately placed in a 0.1 M PB solution containing 0.1% sodium azide (pH 7.4). Every third section was selected for each stain (i.e., SNc TH and IBA1).

For SNc TH staining, sections were washed in phosphate-buffered saline (PBS) (pH 7.4) three times for 5 min each, followed by a 30-min incubation in 0.3% hydrogen peroxide in PBS. Slices were then blocked and incubated overnight in primary antibody solution (5% NGS, 0.3% triton-X, 0.3% bovine serum albumin (BSA) in 0.1 M PBS) with 1:2000 anti-mouse TH (Immunostar, Hudson, WI). The following sections were washed and antibodies in secondary solution (1.6% NGS, 0.16% Triton X, 0.3% BSA, in 0.1 M PBS) were applied to SNc (anti-mouse HRP; 1:200) sections for 4 h. DAB-stained TH+ cells were analyzed using the optical fractionator workflow in Stereo-investigator (MBF, Williston, VT, USA) as we previously used [46]. Six-eight slices were counted beginning at bregma level − 3.08 to the end of the SNc as identified through methods previously described [47]. The SNc was manually traced and then sections were counted by a blind observer under a 63× oil immersion lens. Presented data are the stereological estimate of total SNc dopaminergic neurons.

To label IBA1, sections were washed and blocked and then placed in anti-rabbit IBA1 (Abcam, Cambridge, MA) at a dilution of 1:1000 in primary solution (5% NGS, 0.3% triton-X, 0.3% BSA in 0.1 M PBS for a period of 2 h. Sections were then washed and reacted with either 1:1000 of anti-goat Alexafluor 594 or 647 antibody in primary solution (5% NGS, 0.3% triton-X, 0.3% BSA in 0.1 M PBS). The signal was visualized with immunofluorescence microscopy using Microbrightfield image acquisition software on a Zeiss axioimager2 microscope. All sections were selected and compared between animals at the same distance from bregma.

### Statistical analysis

All data were analyzed by 3 (Water vs. DSS vs. VSL#3) X 2 (Saline vs. LPS and Paraquat) two-way ANOVA with significant interactions further analyzed by means Bonferroni follow-up comparisons (*p* < 0.05) where appropriate. Additionally, analysis of total home cage locomotor activity, and sickness scores was completed using appropriate repeated measures ANOVA’s followed by post hoc analysis. Data is presented in the form of mean ± standard error mean (mean ± SEM). All data was analyzed using the statistical software StatView (version 6.0), and differences were considered statistically significant when *p* < 0.05.

## Results

### DSS significantly altered gut microbiota composition but LPS and paraquat combination treatment did not

**Fig. 2:**
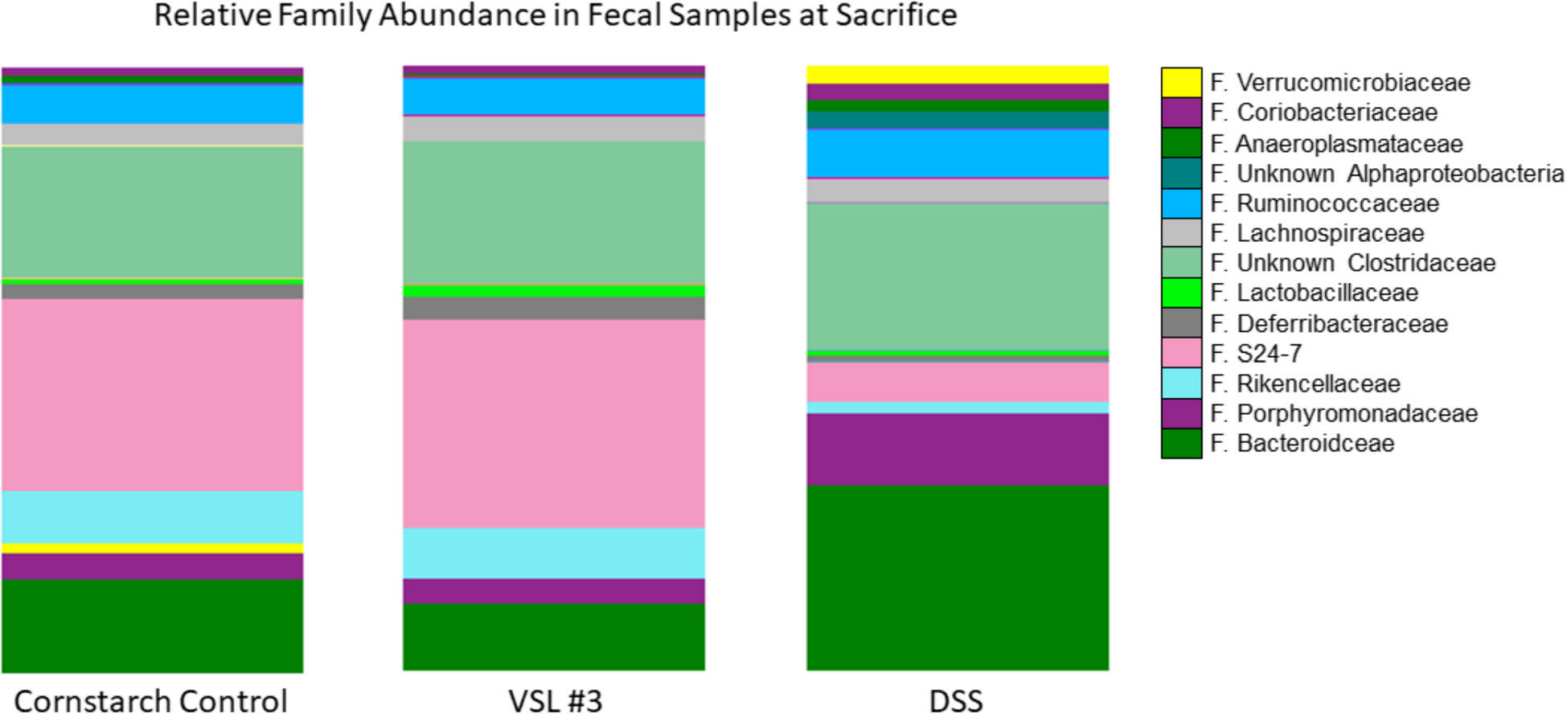
Microbiota sequencing revealed that LPS and paraquat treatment did not alter the composition of the gut microbiome. However, VSL#3 administration increased levels of Streptococcaceae family bacteria but had no other impact, whereas DSS treatment had the biggest effects. Specifically, DSS significantly increased levels of the Verrucomicrobaceae, Bacteriodaceae, Clostridiaceae, and Porphyromonadaceae families and decreased levels of the S24-7 and Rikencellaceae families, relative to cornstarch-treated controls

V4-V5 sequencing was carried out on fresh fecal samples collected at animal arrival to facility and immediately prior to sacrifice. DSS treatment significantly altered microbial composition of the gut, relative to cornstarch controls (Fig. 2). Notably, there was a significant decrease in Rikkencellaceae (*p* < 0.01) and S24-7 (*p* < 0.001). However, DSS increased the proportion of the following families: Bacteroidaceae (*p* < 0.001), Porphyromanadaceae (*p* < 0.001), Verrucomicrobiaceae (*p* < 0.001), and unclassified Clostridaceae (*p* = 0.036). The VSL #3 administration also induced an increase in Streptococcaceae family bacteria (*p* < 0.001); however, no statistically significant effects on Lactobacillaceae or Bifidobacteriaceae. No statistical differences were observed at the class, order, or family level following the LPS and paraquat treatment.

### DSS led to transient weight loss and decreased survival, whereas VSL #3 prevented the LPS and paraquat-induced reduced weight gain

**Fig. 3:**
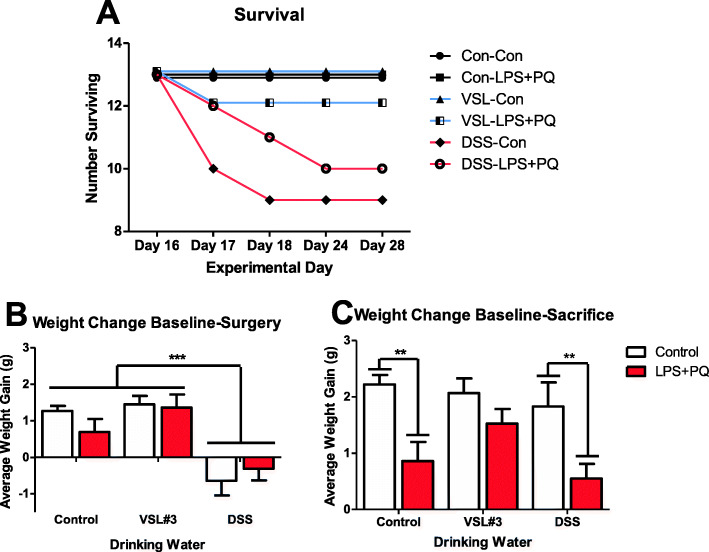
Weight changes and survival throughout the experiment. **a** DSS treatment (red lines with either saline control (Con) or LPS-paraquat co-administration) caused marked sickness and mice reached endpoint in 7 days following cessation of DSS treatment (25–30% reached endpoint). **b** DSS-treated animals lost weight 48 h after cessation of DSS treatment (pre-surgery weight) (*p* < 0.001) and **c** by the end of the experiment, the VSL#3-treated animals recovered, while LPS and paraquat treatment reduced weight gain in control and DSS administered mice (*p* < 0.01). **p < 0.01 compared to controls. ***p < 0.001 compared to other water treatment groups

Over the duration of the experiment, DSS-treated mice displayed considerable signs of illness and reached endpoint significantly more often than non-DSS-treated animals (Fig. 3). Following 5 days of DSS treatment and 2 days of washout, the DSS-treated animals also displayed significant weight loss compared to baseline (*F(2,77) = 23.012, p < 0.001*) (Fig. 3). By the time of sacrifice, there were significant differences in weight that varied as a function of the LPS-paraquat × VSL treatments (*F(2,70) = 3.203, p < 0.05)*. Specifically, the LPS- and paraquat-treated mice lost considerably more weight than controls (*p < 0.05*), but this effect was diminished by the VSL #3 treatment (*p* < 0.05) (Fig. 3).

### DSS affected motor coordination and home-cage activity

**Fig. 4:**
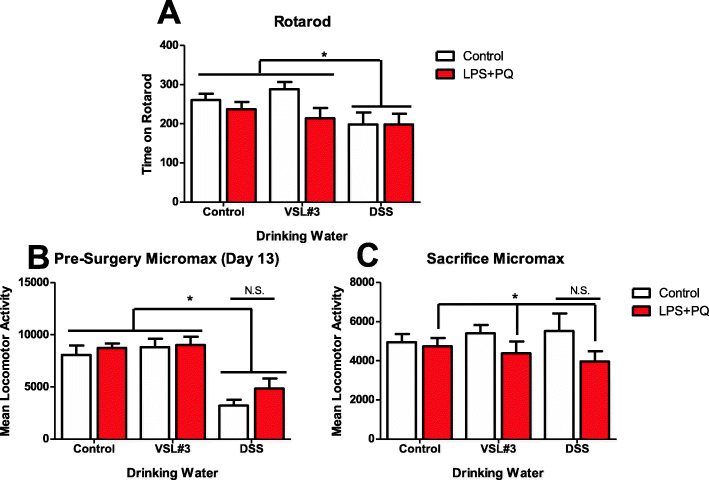
Behavioral motor disturbances were observed in the DSS and LPS + paraquat-treated animals. As shown in panel **a,** all DSS-treated mice exhibited reduced time spent on rotarod, relative to all other animals. Panel **b** illustrates that the DSS treatment initially (prior to LPS and paraquat) reduced home-cage locomotor activity. Thereafter, panel **c** illustrates that by the time of sacrifice, the home-cage locomotor activity was significantly diminished by the LPS-paraquat treatment. However, this decrement was most apparent in the mice that also received DSS earlier. *p < 0.05 compared to non-treated control mice. ***p < 0.001 compared to other water treatment groups

The average time spent on the rotarod apparatus following three discrete trials was significantly affected by the treatment mice had in their water (*F(2,77) = 4.997, p < 0.01*) (Fig. 4a). Specifically, DSS treatment reduced the time that mice were able to stay on the rotating drum (*p < 0.05*). The DSS treatment was also found to reduce overnight home cage locomotor activity as measured by a MMx beam break apparatus (*F(2,80) = 23.06, p < 0.001*) (Fig. 4b). By the final paraquat injection, no DSS effect was observed; however, the LPS and paraquat combination treatments overall now did reduce home-cage locomotor activity at this final test point *(F(1,67) = 4.413, p < 0.05*) (Fig. 4c).

### LPS and paraquat combination treatment reduces TH+ cell count in the SNc

**Fig. 5:**
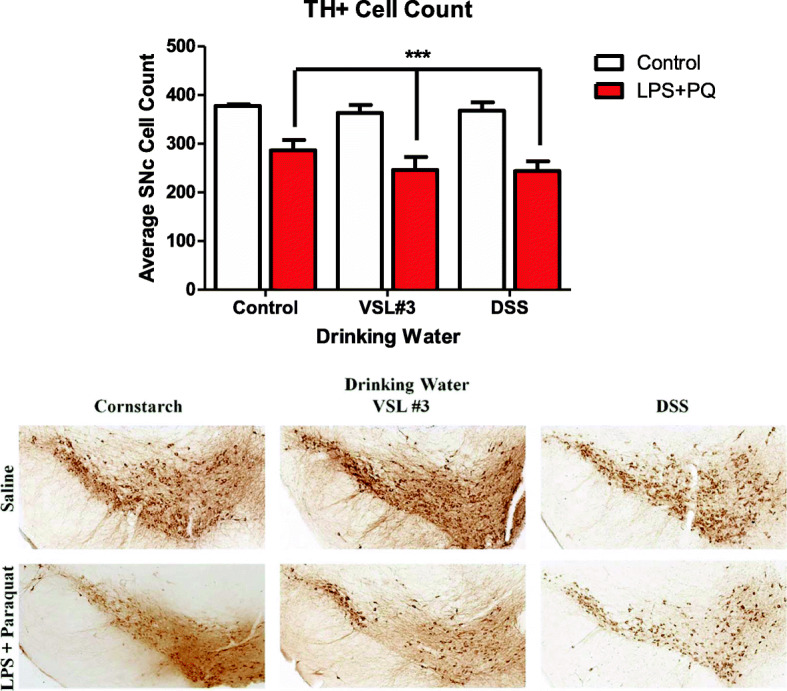
Stereological counts of TH+ neurons in the SNc ipsilateral to the intra-nigral LPS infusion. Clearly, the LPS infusion coupled with systemic paraquat (i.p. 10 mg/kg, six injections over 2 weeks) administration significantly reduced the number of viable TH+ neurons within the SNc. But no significant differences whatsoever were observed concerning the VSL#3 and DSS treatments. ***p < 0.001 compared to saline-treated animals

Stereological assessments revealed that the LPS and paraquat treatment affected neuronal TH+ counts in SNc (*F(1,18) = 50.539, p < 0.001*) (Fig. 5). Specifically, all mice that received LPS and paraquat administration had a reduced number of surviving TH+ dopaminergic neurons within the SNc (*p* < 0.05). This effect, however, was not significantly influenced by either the DSS or the VSL #3 treatments.

### DSS treatment further augmented LPS-paraquat-induced microglial activation in the SNc

**Fig. 6:**
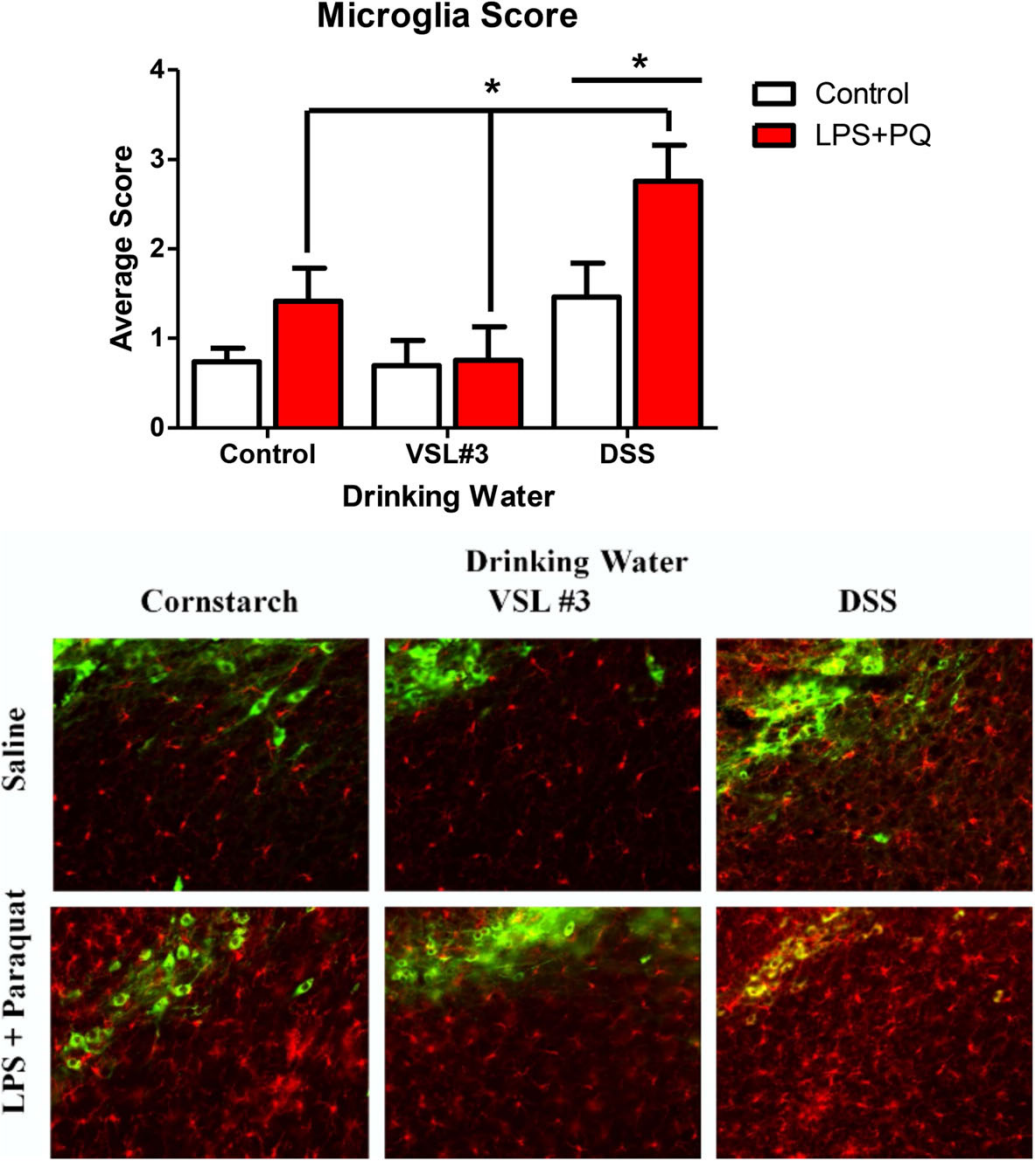
Microglial activation was assessed using a validated semi-quantitative rating scale for morphology on ×20 images of SNc sections with IBA-1 (red) and TH (green) immunofluorescence. The DSS treatment alone significantly increased ratings of activation scoring (*p* < 0.05), as did the LPS and paraquat combination treatment (*p* < 0.001). But the VSL#3 treatment was without significant effect on microglial morphological ratings. *p < 0.05 compared to saline treated animals ***p < 0.001 compared to either: **a** control water-treated mice that received LPS + PQ infusion or **b** saline-infused mice that received DSS treatment

Microglia morphological ratings that were scored by an experimentally blinded researcher revealed that both the LPS plus paraquat and DSS treatments each significantly increased microglial morphological ratings (*F(1,27)*, *(2,27) = 7.052*, and *10.07*, respectively, *p < 0.05 for significant main effects*) (Fig. 6). However, post hoc comparisons revealed that the largest effect was apparent in mice that received the combination of both the DSS pre-treatment followed by LPS and paraquat, relative to those that only received one of these treatments (*p* < 0.05). For the VSL3# treatment, however, there was no significant main effect or interaction

### DSS treatment increased intestinal TNF-α and IL-1β mRNA expression

**Fig. 7:**
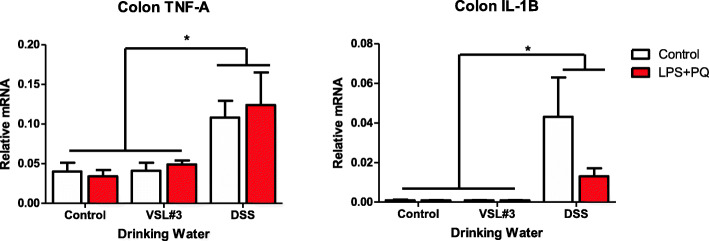
Pro-inflammatory cytokine expression in the colon was quantified by qRT PCR and normalized against GAPDH expression. Both TNF-α and IL-1β levels were found to be elevated in DSS-treated mice following sacrifice. *p < 0.05 compared to water intake control animals

Oral administration of the DSS resulted in lasting pro-inflammatory cytokine mRNA alterations in the colon. Notably, DSS treatment increased both TNF-α mRNA (*F(2,27) = 6.297*, *p < 0.01*) (Fig. 7a) and IL-1β mRNA (*F(2,27) = 4.060*, *p = 0.02*) (Fig. 7b), relative to the non-DSS-treated animals. There was no effect of VSL#3, and similarly, the only effect of LPS plus paraquat was a paradoxical reduction in IL-1β in DSS-treated mice, compared to the saline-treated DSS mice (*p* < 0.05)

### Circulating immune factors were increased by LPS and paraquat combination treatment, as well as by DSS administration

**Fig. 8:**
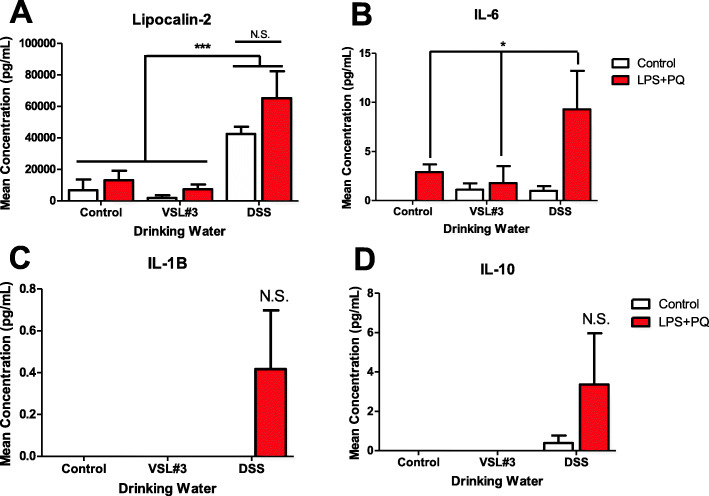
As shown in panel **a**, plasma lipocalin-2 **(**LCN-2) levels were significantly elevated by DSS treatment, whereas panel **b** shows that circulating IL-6 levels significantly increased in the LPS- and paraquat-treated mice. Both IL-1β (*p* = 0.30; panel **c**) and IL-10 (*p* = 0.26; panel **d**) levels were modestly but not significantly elevated in DSS-treated mice that also received LPS + paraquat. *p < 0.05 compared to control animals, ***p < 0.001 compared to other water treatment groups

The plasma level of lipocalin-2 (LCN2), a neutrophil activation marker, was found to be increased by DSS administration (*F(2,22) = 13.881*, *p < 0.001*) (Fig. 8a), but unaffected by the LPS and paraquat or VSL#3 treatment. The LPS and paraquat treatment also increased levels of circulating IL-6 (*F(1,24) = 4.791*, *p = 0.03*) (Fig. 8b), and this effect was particularly evident in the LPS-paraquat mice that were also treated with DSS, such that levels in this group exceeded all other animals (*p* < 0.05). However, levels of IL-1β and IL-10 showed no significant alterations with any treatment, albeit there was a variable increase again in the DSS and LPS plus paraquat treated mice (Fig. 8c, d).

### LPS and paraquat administration altered proteins involved in inflammation, astrogliosis, and cell motility

**Fig. 9:**
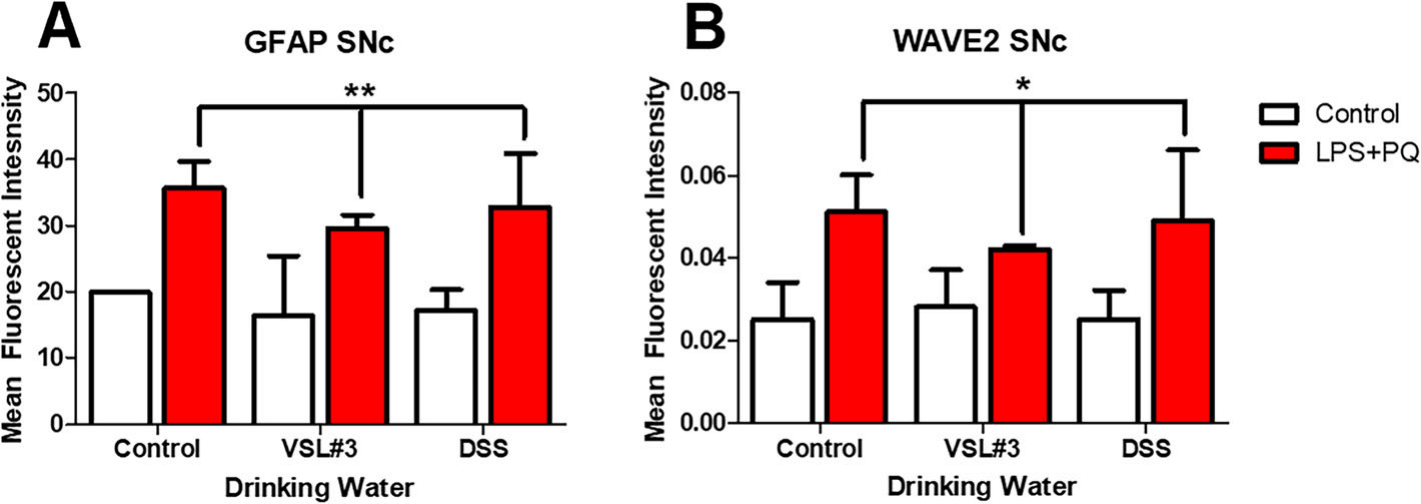
Western blot assessment of SNc tissue revealed increased levels of GFAP (**a**) and WAVE2 (**b**) in LPS and paraquat-treated mice. *p < 0.05 compared to saline-treated animals, **p < 0.01 compared to control animals

Irrespective of VSL#3 and DSS treatment, GFAP, an astrocyte marker, was found to be significantly upregulated by LPS and paraquat treatment (*F(1,16) = 11.134*, *p < 0.01*) (Fig. 9a) and similarly, WAVE2, a marker of actin cytoskeleton remodeling, was also upregulated by the same treatment (*F(1,16) = 5.661*, *p < 0.03*) (Fig. 9b).

## Discussion

Much recent evidence has focused on a role for the gut microbiota in neurological diseases, including PD. Indeed, Scheperjans et al. became the first to publish human PD data supporting an alteration of gut bacterial species in PD patients compared to age-matched controls [16]. Several studies have followed this in different populations confirming that these alterations are fairly consistent across cultural and ethnic borders [15, 25, 48, 49]. Even more strikingly, direct transfer of a microbiota from human PD patients (compared to that from matched non-PD controls) provoked behavioral and neuroinflammatory consequences in α-synuclein overexpressing mice [72]. Despite these findings, it is still unknown whether gut microbiome changes precede or contribute to the disease. Similarly, it is unclear whether the gastrointestinal symptoms which typically are displayed prior to motor symptoms in PD patients may drive these microbial alterations. That said, a recent study did find that seeding the duodenal wall with α-synuclein fibrils in mice resulted in a pathological spread of the protein, which is consistent with the idea of gastrointestinal prodromal state contributing to later PD pathology [74].

To assess for potential protective or predisposing effects of alterations in the gut microbiome, we induced PD-like pathology (using intra-SNc LPS infusion followed by paraquat injections) in mice that were previously treated with either DSS, a toxin commonly used to model colitis, or VSL #3, a probiotic previously shown to have anti-inflammatory consequences. We found that DSS markedly altered the composition of gut microbiota, whereas neither VSL#3 nor the LPS-paraquat treatments had any observable consequences (at least, not at the time of sacrifice). Neither the DSS nor the VSL#3 influenced the loss of SNc dopaminergic neurons that was induced by LPS plus paraquat; however, the DSS treatment influenced inflammatory factors and aspects of the microglial state. Moreover, the VSL#3 treatment did diminish the weight loss that was promoted by LPS and paraquat.

Data on modulating the gut microbiota may be key to understanding the factors that lead to the development of PD for two key reasons: (1) If altered gut microbiota can predispose individuals towards PD later in life, it may be possible to identify at risk individuals prior to neuronal degeneration and (2) given the relative ease at which bacteria can be introduced to the gut, there may be opportunity for prophylactic pre- or pro-biotic treatment and similarly, the ease at which samples from the gut can be obtained from potential patients is important [50, 51].

VSL #3 is commonly prescribed for individuals suffering from ulcerative colitis and irritable bowel syndrome, and it can limit peripheral inflammation by decreasing intestinal permeability. Several studies have found beneficial effects of VSL #3 treatment in murine models [29, 30, 52]. Intriguingly, one study that examined the reaction to VSL#3 in healthy C57BL/6 mice found no beneficial alterations, but quite a marked upregulation of the fractalkine receptor, coupled with decreased T cell expression [53]. Our lack of strong anti-inflammatory changes following VSL#3 treatment may be related to timing and dose of the probiotic, and future studies will be required using more robust dose-response experiments.

Yet, the fact that the VSL#3 treatment did blunt the weight loss evident following LPS and paraquat exposure, suggests that it was sufficient to at least influence gross clinical aspects of the induced inflammatory response. The VSL#3 treatment also tended to diminish immunofluorescent ratings of microglial morphology, raising the possibility of a central role in inflammatory state, but this effect was not statistically significant. Overall, the impact of the probiotic was mostly not evident, especially when compared to the marked effects of DSS and LPS plus paraquat.

Our presently administered 5.4 × 10^9^ CFU dose of VSL#3 per day is not unlike previous studies that used a 4 × 10^9^ CFU dose (daily over a 7-day interval) but reported beneficial effects in mice that had 2,4,6-trinitrobenzene sulfonic acid-induced colitis [40]. Others have utilized a lower dose of 3 × 10^8^ CFU VSL#3 for a period of 28 days, but human studies actually use doses that are higher in the range of 4–9 × 10^11^ CFU [39]. Importantly, however, these previous animal studies utilized oral gavage as a means of VSL#3 delivery [39], whereas we utilized the non-stressful approach of delivering VSL#3 in the drinking water. Hence, the dosage and rapidity of delivery on probiotics might affect their efficacy and it is possible that delivering the probiotic in a more direct (albeit stressful) gavage route might cause more robust variations in the microbiome.

Future studies might do well to consider the possibility of varying the (a) timing of VSL#3 exposure, (b) the animal strain used, and (c) the type of model of PD-like pathology. Indeed, a recent meta-analysis found that VSL#3 was effective in preventing relapse in ulcerative colitis patients that presently had a quiescent disease state, but was not effective for inducing remission in active cases or for preventing relapse in post-operative cases [54]. The genetic background of the animal employed is also important, with the presently used C57Bl6/J mice used typically display a biased towards inflammatory Th1 immune responses. It was in fact shown that BALB/c and C57BL/6 mouse strains showed differences in gene expression in the colon and small intestine following VSL#3 treatment [39]. Further, differences in inflammatory factor expression between these strains are larger than the actual impact of VSL#3 and similar larger inter-individual differences were also apparent in human subjects [39]. Finally, animal models of PD that involve genetic vulnerability factors, such as LRRK2, PINK1, or DJ-1, might yield different sensitivity to probiotics. Indeed, these genes are known to influence inflammatory processes and have been associated with variations in the gut microbiome [55]. Unlike the present model, using animal models that focus on early stages of disease before pathology takes root or is very severe may be useful. In this regard, it is likely that the potential success of probiotic treatments might be dependent upon addressing pathology during the early stages of disease when major lipid and protein alterations might be first driving inflammatory and oxidative stress [56, 57].

In contrast to VSL#3, DSS substantially altered gut microbiota composition following treatment, similar to previously reported data [58, 59]. Most notable was a drastic increase in the Bacteriodaceae family and a decrease in the S24-7 family. These microbial changes may contribute to local inflammatory changes since previous studies showed that DSS-induced pathology can be limited by modification or transplant of certain such gut microbes [60]. DSS further provoked an increase in IL-1b and TNF-α expression in the colon, along with robust elevations of circulating neutrophils and IL-6 levels. Interestingly, these changes were paralleled by increased morphological ratings of SNc microglia, which is consistent with a role for DSS in promoting heterogeneous neuroinflammation in the CNS [61]. Accordingly, a just published study found that DSS induced the differential expression of numerous genes within the striatum that might be important for PD, including those involved in immune processes and cellular detoxification [62]. Accompanying these alterations, we found mice to display acute sickness symptoms characterized by rapid weight loss, diarrhea (albeit this measure was subjectively noted and not quantified), and reduced locomotor activity similar to previous experiments using the same model [63]. These effects were quite severe and led to a proportion of animals reaching humane endpoint. Surviving animals did, however, recover and did not show any lasting behavioral responses at time of sacrifice, but they did display decreased coordination on the rotarod test at this time. It seems that DSS provoked marked peripheral and central immune changes that may be indicative of a widespread innate inflammatory reaction that caused transient sickness, followed by longer term motor disturbances. It is likely that local gut inflammatory processes (associated with microbiome alterations) and enhanced “leakiness” of gastric membranes that were induced by the DSS treatment acted as an initial source of the widespread pathology observed. It could further be envisioned that stress/toxin-induced gastric disturbances could act as a primary driver of systemic inflammatory processes and eventually, disturb CNS processes possibly giving rise to motor, cognitive, and mood changes. These effects of DSS are of particular interest to note in relation to PD-linked toxins that likewise induce transient illness, as well as co-morbid signs of anxiety and/or depressive-like symptoms [75].

Although one potential mediator of gut-brain communication is neural fibers such as the vagal nerve [12], much evidence suggest the importance of cytokines and other soluble inflammatory factors that normally regulate innate immunity. To this end, DSS treatment increased circulating levels of the neutrophil marker, LCN2, which is consistent with previous reports showing elevations in plasma and feces [63, 64]. Although LPS plus paraquat treatment did not affect neutrophil levels, it did further augment the rise observed with DSS treatment. It was also observed that IL-6 increased following LPS plus paraquat administration, as we and others have previously reported [45, 65]. However, no effect of DSS was apparent, contrary to previous reports in the gut [66]. In addition, non-significant elevations of IL-1b and IL-10 were apparent in mice that received both DSS pre-treatment and subsequent LPS and paraquat exposure.

LPS and paraquat treatment have previously been shown to induce microglial activation in the SNc [67], and we presently observed that DSS pre-treatment enhanced this activation. Despite this, SNc dopaminergic neuronal loss was not influenced by either DSS or VSL#3, which is surprising given the evidence linking microglial state to neurodegeneration [65, 68–70]. Yet, we found that LPS plus paraquat also induced increase in astroglia marker (at least a subset of astrocytes, as determined by GFAP) and this was unaffected by the DSS treatments. These cells could be of importance to the present outcomes given that there is a large literature suggesting a role for astrogliosis in PD [73]. In any case, it was interesting that microglia appear more influenced or tied to the DSS-induced gut changes than were astrocytes. Additionally, short chain fatty acids (SCFA) are another mechanism through which the microbiota of the gut can affect the brain and in particular, these may mediate many of the effects of the gut on central microglial cells. For instance, administering SCFAs corrected the alterations in microglial gene expression provoked by an absence of microbiota in germ-free mice or those that were administered antibiotics [71]. Also, while raising α-synuclein overexpressing mice in germ-free conditions ameliorated the usual motor deficit evident, the administration of SCFAs restored the behavioral pathology [72].

## Conclusions

In brief, we found that DSS-induced variations in microbiota were associated with an augmented inflammatory profile, but did not influence toxin-provoked dopaminergic neurodegeneration. DSS also had motoric effects but this may have stemmed from the obvious signs of illness associated with the treatment. Probiotic VSL#3 did not influence any PD-like outcomes and appeared to have minimal consequences overall. These data are novel and speak to the possible connection between gut dysbiosis and PD development. Indeed, the gastric disturbances that are common in PD might be related to microbiome and inflammatory changes, possibly akin to those presently observed. Yet, our model at least does not support any contention that such gut alterations have the ability to directly modify nigral degeneration. Of course, alternate models, such as those employing a-synuclein fibrils or aggregates, may yield a different outcome. In fact, there is reason to suppose that, unlike our toxin-based LPS plus paraquat model, a-synuclein accumulation in the gut might directly or indirectly affect midbrain dopaminergic functioning [13, 23].

Whatever the case, our data overall do not support a role for the microbiota in modulating frank dopaminergic neurodegeneration. However, they do suggest that alterations in the microbiome may occur prior to disease onset and could contribute or at least modify the inflammatory state often observed in PD patients.

## Data Availability

All data supporting the conclusions of this article will be included with this article.

## Abbreviations

DSS: Dextran sodium sulphate
KO: Knockout
LPS: Lipopolysaccharide
LCN2: Lipocalin
MMx: Micromax
PD: Parkinson’s disease
PQ: Paraquat
